# O'nyong nyong Virus Molecular Determinants of Unique Vector Specificity Reside in Non-Structural Protein 3

**DOI:** 10.1371/journal.pntd.0001931

**Published:** 2013-01-24

**Authors:** Kali D. Saxton-Shaw, Jeremy P. Ledermann, Erin M. Borland, Janae L. Stovall, Eric C. Mossel, Amber J. Singh, Jeffrey Wilusz, Ann M. Powers

**Affiliations:** 1 Division of Vector Borne Infectious Diseases, Centers for Disease Control and Prevention, Fort Collins, Colorado, United States of America; 2 Department of Microbiology, Immunology and Pathology, Colorado State University, Fort Collins, Colorado, United States of America; University of California, Berkeley, United States of America

## Abstract

O'nyong nyong virus (ONNV) and Chikungunya virus (CHIKV) are two closely related alphaviruses with very different infection patterns in the mosquito, *Anopheles gambiae*. ONNV is the only alphavirus transmitted by anopheline mosquitoes, but specific molecular determinants of infection of this unique vector specificity remain unidentified. Fifteen distinct chimeric viruses were constructed to evaluate both structural and non-structural regions of the genome and infection patterns were determined through artificial infectious feeds in *An. gambiae* with each of these chimeras. Only one region, non-structural protein 3 (nsP3), was sufficient to up-regulate infection to rates similar to those seen with parental ONNV. When ONNV non-structural protein 3 (nsP3) replaced nsP3 from CHIKV virus in one of the chimeric viruses, infection rates in *An. gambiae* went from 0% to 63.5%. No other single gene or viral region addition was able to restore infection rates. Thus, we have shown that a non-structural genome element involved in viral replication is a major element involved in ONNV's unique vector specificity.

## Author Summary

O'nyong nyong virus (ONNV) is unique in that it is the only alphavirus, and one of few viruses in general, to be transmitted to humans by the bite of an anopheline mosquito. The genetics responsible for this unique vector specificity would be useful information in helping to develop antivirals, vaccines, and other methods for interrupting virus transmission. Previous research using other arboviruses has shown that specific viral genomic regions, amino acid sequences, or even single nucleotide mutations can have a profound effect on virus growth, infection, and virulence characteristics. Using chimeric viruses that substitute a gene from one virus with a gene from a closely related virus is a proven method of evaluating the relative contribution of each gene to a given phenotype. Our study analyzed both structural and non-structural regions of the ONNV genome using chimeric viruses and artificially infected *Anopheles gambiae* mosquitoes. When ONNV non-structural protein 3 (nsP3) replaced nsP3 from chikungunya virus in one of the chimeric viruses, infection rates in *An. gambiae* went from 0% to 63.5%. No other single gene or viral region addition was able to restore infection rates. That ONNV nsP3 is largely responsible for ONNV's unique ability to infect *An. gambiae* is especially interesting since the exact mechanisms and functions of this highly-variable protein remain poorly understood.

## Introduction

O'nyong nyong virus (ONNV) is an arthropod-borne virus (arbovirus) associated with a small number of large- scale epidemics. One such epidemic began in 1959 in Uganda, lasted three years and affected over 2 million people [1]. Serological evidence of ONNV transmission indicated circulation in Kenya until the late 1960s [2], additional serological surveys in 1974–1975 showed circulation in West Africa [3], but ONNV did not cause another epidemic until 1996, when 400 people were sickened in the Rakai district in southern Uganda [4]. The known distribution of ONNV mirrors that of the mosquito vectors that transmit the virus, *Anopheles gambiae* and *Anopheles funestus* [5]. Humans are the only currently known reservoir of ONNV [6]. ONNV infection in humans is usually self-limiting, but does cause a low grade-fever, joint pain, lymphadenopathy, and a generalized papular or maculopapular rash [7].

Chikungunya (CHIKV) virus is a closely related alphavirus which has caused millions of cases of disease throughout countries in and surrounding the Indian Ocean since its re-emergence in 2004 [8]–[10]. Additional cases occurred in travelers returning from affected areas to Asia, North America, and to Europe, where a few small epidemics have since occurred due to autochthonous transmission [11]–[14]. Humans are infected with CHIKV when bitten by infected *Aedes aegypti* or, during epidemics, *Aedes albopictus* mosquitoes. Patients infected with CHIKV suffer from clinical symptoms similar to those infected with ONNV except that the fever is a higher, there is typically no lymphadenopathy, and the arthralgia is both incapacitating and chronic.

CHIKV and ONNV diverged from a common ancestor thousands of years ago [15] and despite their genetic similarity, ONNV and CHIKV have distinct ecologies. In particular, they are not transmitted by the same mosquito vectors [6]. In fact, ONNV is the only alphavirus to be vectored by anopheline mosquitoes [5] while CHIKV is transmitted by culicine mosquitoes. The differential mosquito infectivities of CHIKV and ONNV have been characterized in the laboratory where *An. gambiae* mosquitoes have been shown to be highly susceptible to ONNV infection and refractory to CHIKV infection [6]. Thus, this mosquito serves as an ideal system to examine the molecular determinants of infection using hybrids of the two viruses.

Members of the genus *Alphavirus* contain a single-stranded, positive-sense RNA genome (∼11.7 kb) that contains two regions, a non-structural domain making up the 5′ two-thirds of the RNA and a structural domain at the 3′ end making up the remaining one-third of the genome [16]. The non-structural domain encodes four viral non-structural proteins: nsP1, nsP2, nsP3, and nsP4 which are essential for replication and polyprotein processing. In addition to copying the RNA genome, the non-structural proteins synthesize 26S subgenomic mRNA which is capped and polyadenylated and which ultimately produces five individual structural proteins (capsid, E3, E2, 6K, E1) [17]. Previous studies with chimeric alphaviruses have shown that viral components in both the structural and non-structural portions of the Venezuelan equine encephalitis genome contribute to infection phenotypes in guinea pigs [18]. Similar results were seen with chimeric eastern equine encephalitis viruses, with both structural and nonstructural proteins contributing to neurovirulence and viral tissue tropism in mice [19]. Chimeric viruses are also useful for studying virus-vector interactions as seen in a study that mapped mosquito infection determinants specifically to the E2 envelope glycoprotein region of Venezuelan equine encephalitis [20]. Earlier studies using chimeric ONNV suggested that all of the viral structural proteins are necessary for ONNV to infect *An. gambiae* mosquitoes [21]; however these studies used limited sample sizes and looked only at the structural regions of the genome. The current study expanded upon this earlier work to examine the contribution of each specific viral region or gene to virus-vector specificity.

## Methods

### Infectious clone production

**Figure 1:**
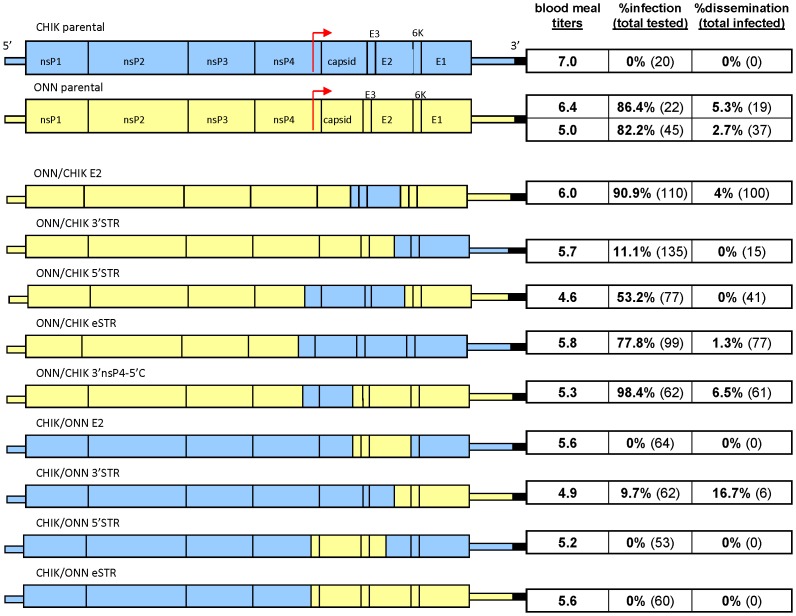
Infection rates with schematic diagrams. Infection rates at day 8 post-infection with parental and structural-region chimeric viruses in *An. gambiae*. Results from the 2–3 replicate feeds were combined and the titer (log_10_ PFU/ml) shown is an average (for titers that were within 0.5 log_10_ PFU/ml of one another). Infection rate is determined using mosquito bodies while dissemination rate is derived from heads.

**Figure 2:**
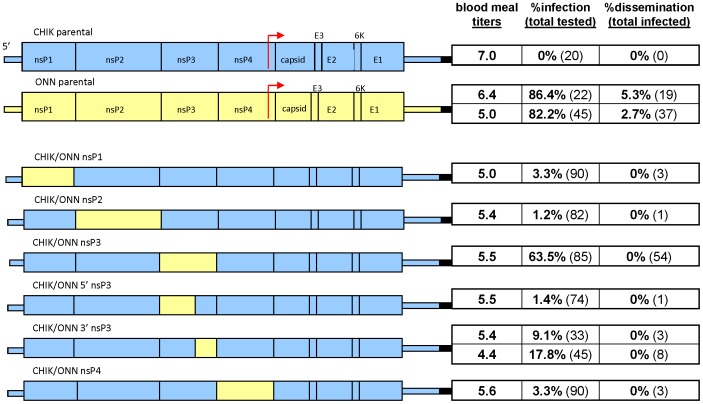
Infection rates with schematic diagrams. Infection rates at day 8 post-infection with parental and non-structural-region chimeric viruses in *An. gambiae*. Results are reported as in Figure 1. Infection rate is determined using mosquito bodies while dissemination rate is derived from heads.

All plasmid clones used in this study were designed and constructed in house. The full length clone pONN.AP3 was constructed from ONNV strain SG650 [5] (GenBank accession number AF079456) while pCHIK.b was constructed from CHIKV strain 37997 (GenBank accession number AY726732). These two full –length parental clones were used to construct 15 chimeric viruses as shown in Figures 1 and 2.

All plasmid clones designed to evaluate structural regions of the genome were constructed in a similar fashion, with the substituted region produced from a PCR product and the backbone region produced from the parental plasmid clones described previously. For example, to construct pCHIK/ONN E2, the E2 region of ONNV was amplified from parental pONN.AP3 by PCR with PFU turbo polymerase (Stratagene, La Jolla, CA). The ONNV amplicon and pCHIK.B were digested with the same restriction enzymes (Table 1). When necessary, appropriate restriction enzyme sites were added to pCHIK.B using a QuikChange XL site-directed mutagenesis kit (Stratagene) according to the manufacturer's instructions. Modifications were performed so that no amino acid changes were introduced and all viruses generated from constructs with introduced mutations were tested to insure they replicated in a manner comparable to the parental viruses. Both restriction digests were run on an agarose gel at low voltage for at least 8 hours. The doubly-digested insert and plasmid backbone were cut from the gel and purified from the agarose using the MinElute Gel Extraction Kit (Qiagen, Valencia, CA). The backbone vector was treated with Antarctic Phosphatase (NEB, Beverly, MA) to remove the 5′ phosphate groups, thus preventing self- ligation of the plasmid. Prepared plasmid backbone and insert were ligated overnight with T4 DNA ligase (NEB) and then electroporated into XL-1 Blue electrocompetent cells (Stratagene). Transformed cells were grown on YT plates with 50 mg/ml ampicillin and incubated overnight at 37°C. Colonies were picked and screened for confirmation of ONNV insert by PCR. Plasmid clones were confirmed by sequencing the entire construct.

**Table 1:** Restriction enzyme sites used to create infectious plasmid clones.

Chimeric virus	region swapped	restriction enzyme used
Onn/Chik E2	nt 8349–9873	AvrII, AvrII
Onn/Chik 3′STR	nt 9545–12005	BamHI, NotI
Onn/Chik 5′STR	nt 7267–9568	ClaI, BamHI
Onn/Chik eSTR	nt 7267–12028	ClaI NotI
Onn/Chik 3′nsP4-5′C	nt 7267–8372	ClaI (engineered), AvrII
Chik/Onn E2	nt 8253–9777	AvrII, AvrII
Chik/Onn 3′STR	nt 9343–11754	SdaI (engineered), NotI
Chik/Onn 5′STR	nt 7148–9320	ClaI, SdaI (both engineered)
Chik/Onn eSTR	nt 7148–11731	ClaI (engineered), NotI
Chik/Onn nsP1	nt 78–1682	type II enzyme, precise substitution
Chik/Onn nsP2	nt 1683–4076	type II enzyme, precise substitution
Chik/Onn nsP3	nt 4077–5765	type II enzyme, precise substitution
Chik/Onn 5′ nsP3	nt 4077–5017	type II enzyme, precise substitution
Chik/Onn 3′ nsP3	nt 5017–5768	type II enzyme, precise substitution
Chik/Onn nsP4	nt 5649–7499	type II enzyme, precise substitution

Plasmid clones designed to evaluate non-structural regions of the genome were engineered as exact gene substitutions by using a series of subclones, as described in the Protocol S1 and in Figures S1, S2, S3, S4, S5, S6. Briefly, CHIKV regions flanking the desired non-structural protein, and containing convenient restriction sites were amplified from pCHIK.b by PCR with PFU turbo polymerase (Stratagene). Primers used for amplification added a type II restriction enzyme site to the outside of each desired insert product. These two PCR products and a modified cloning vector, pUC19M2 were each double digested using type I enzymes exterior to the type II engineered sites. A 3-way ligation then produced the first subclone (pUC19M2 with the CHIKV sequence flanking where the desired non-structural protein sequence would subsequently be inserted). The desired ONNV non-structural protein was amplified using primers which added the same type II enzyme site as was added to the PCR products. The first subclone and the ONNV PCR product were both digested with the same type II enzyme, which cuts itself out upon digestion. Ligation of the two digested products produced a second subclone (pUC19M2 with the entire and exact ONNV non-structural protein, flanked by CHIKV sequence). This second subclone and pCHIK.b were then digested using the convenient restriction sites already present in the flanking CHIKV sequence. Ligation of the doubly-digested pCHIK.b backbone and the insert obtained from the second subclone produced the final construct. Colonies were screened and verified by complete genome sequencing and plasmid DNA was prepared as described above.

### Rescue of virus from infectious clone templates

Templates for *in vitro* transcription were generated by linearizing each full-length clone with a unique Not I restriction site present downstream of the poly (A) tail. Linearized plasmids were treated with Proteinase K (Invitrogen, Carlsbad, CA) to digest any endogenous RNases or DNases. DNA was purified using a phenol-chloroform extraction followed by an ethanol precipitation. A 20 µl aliquot of linearized and treated DNA was then transcribed *in vitro* by incubating the DNA with 0.4 µl (100 mM) each rNTP (Promega, Madison, WI), 0.4 µl BSA (10 mg/ml) (NEB, Beverly, MA), 2 µl DTT (100 mM) (Promega, Madison, WI), 8 µl (5x) transcription buffer (Promega, Madison, WI), 1.3 µl (15 U/µl) T7 RNA polymerase (Promega, Madison, WI), and 4 µl (10 mM) A-cap-structure analog (NEB, Beverly, MA) for one hour at 39°C. Transcribed RNA (10 µl) was mixed with 400 µl BHK-21 cells (1×10^7^cells/ml) in a 2 mm gap cuvette (BTX:Harvard Apparatus, Inc., Holliston, MA) and electroporated twice using a BTX ElectroCell Manipulator with the following settings: 460volts, 725ohms, 75 µF [22]. After electroporation, the cells were transferred to a T-25 tissue-culture flask. Dulbecco's Modified Eagle Medium (DMEM) with 10% by volume fetal bovine serum and 1% by volume penicillin/streptomycin was added to the flask before incubation at 37°C. Tissue culture supernatant was harvested approximately 72 hours post-transfection or when cytopathic effects (CPE) were observed. Supernatant was aliquoted and stored at −80°C until later use.

### Sequencing

Each time a virus was generated, the entire virus was sequenced to verify fidelity to the original sequence. Viral RNA was extracted using QIAamp Viral RNA Mini Kit (Qiagen). Extracted RNA was then added to a reverse-transcriptase PCR reaction using the Titan One Tube RT-PCR System (Roche, Indianapolis, IN). Complementary DNA for sequencing the 5′ end of each viral genome was generated using a FirstChoice RLM-RACE Kit (Ambion, Austin, TX). This complementary DNA was then sequenced using virus-specific primers with the Big Dyev3.1 kit on an ABI 3130xl genetic analyzer (Applied Biosystems, Foster City, CA). Sequence files were aligned and analyzed for sequence quality and genome coverage using Lasergene suite software (DNASTAR, Madison, WI).

### Titrations

Virus rescued from clones was titered by plaque assay. Ten-fold virus dilutions from 10^−1^ to 10^−7^ were added to individual well of 6-well plates covered in monolayers of VERO cells. Plates were incubated at 37°C, with 5% CO_2_. Cells were fixed 48–72 hours later using a solution of 40% methanol and 0.25% crystal violet in water. Plaques were then counted and titers were calculated as plaque forming units per milliliter (PFU/ml).

### Mosquito infections

The ability of each chimeric virus to infect mosquitoes was evaluated using the G3 strain of *An. gambiae*, originally obtained from the National Institute of Health. This strain has been maintained as a colony in our lab with rearing conditions that include a 12∶12 hour light∶dark cycle in chambers maintained at 28°C with approximately 95% humidity [23].

Infectious blood meals were prepared from equal volumes of packed, calf erythrocytes, 10% sucrose in fetal bovine serum, and 4.4–6_log 10_ PFU/ml of virus. Mosquitoes were allowed to feed on the warmed infectious blood meal for one hour through an artificial membrane feeder (Hemotek, Accrington, UK). Fully engorged females were separated and maintained for an incubation period of up to 12 days. Mosquitoes were sacrificed at days 4, 8, and 11 or 12 post-infectious-blood meal. Heads and bodies were separated into individual tubes and stored at −80°C until subsequent processing. Infection rates were determined using individual bodies and dissemination rates were calculated as the number of positive heads among the positive bodies. At least two replicate infectious feeds were done for each chimeric virus, with replicate feeds performed entirely independent of one another. No less than 140 mosquitoes were tested for any one chimeric virus.

### Mosquito processing

Individual frozen mosquito bodies and heads were triturated in 300 µl of DMEM supplemented with (by volume): 10% heat-inactivated fetal bovine serum, 1% penicillin/streptomycin, 0.1% gentamicin, and 0.1% Fungizone. The mosquito homogenates were passed through a 0.2 µm Gelman Acrodisc filter (Krackeler Scientific Inc., Albany, NY) to remove potential bacterial or fungal contaminates. Filtrate from each body or head was added to a single well of a 96-well flat-bottom tissue-culture plate, along with 50 µl of prepared BHK-21 cell suspension (approximately 4.6 log_10_ cells/well). Inoculated tissue-culture plates were incubated at 37°C for 5 days. Cells were observed daily for CPE due to virus replication. Virus replication in mosquito body samples indicated that virus had infected the mosquito's midgut, while replication in the mosquito heads showed a disseminated infection.

### Confirmation of viral replication

To confirm that all constructed viruses were comparably replication competent, growth curves were performed in cell culture on all rescued viruses. Briefly, 24-well plates (Corning, Corning, NY) were seeded with Vero (African green monkey) cells. Monolayers at 90% confluency were infected with virus at a multiplicity of infection (MOI) of 0.1. At specified times post infection, supernatant was removed from two wells for each virus and placed in a screw-cap cryovial at −70°C until titration by plaque assay. Titration results for each virus were compared at all time points by the student t-test.

To confirm virus replication (and not just persistence of the input virus) within the mosquito, five females that had fed on the ONNV infectious blood meal were sacrificed every other day post-infectious feed. Each body and head was processed separately, as described earlier. RNA was extracted from homogenized mosquitoes using QIAamp Viral RNA Mini Kit (Qiagen). The amount of RNA in each head and body was determined using the Quanti Tect Probe RT-PCR kit (Qiagen) and a TaqMan Real-Time PCR assay as previously described [24], except that ONNV SG650 specific primers were used (10692 FWD- 5′ GCA GGG AGG CCA GGA CAG T, 10840 REV- 5′ GCC CCT TTT TCY TTG AGC CAG TA). The real-time probe was labeled with a 5′ end HEX reporter dye and a 3′ end BHQ1 quencher dye (10759 FWD- 5′ AAA GAC CAG CGG CAG GAG CAA TAC AC) and PCR results are reported here as PFU-equivalents/mosquito by comparison with known concentration standards.

### Statistical significance

Fisher's exact probability test was employed to evaluate whether infection rates with chimeric viruses were statistically different from those with parental viruses. The infection rate was defined as significantly different from parental CHIKV if the two-tailed p-value was <0.007. The two-tailed p-value had to be <0.01 to be statistically different from parental ONNV infection rates. Both adjusted alphas were obtained using the Bonferroni correction for multiple comparisons to ensure an overall Type I error of 0.05. Computations were made using freely-available software [25].

## Results

**Figure 3:**
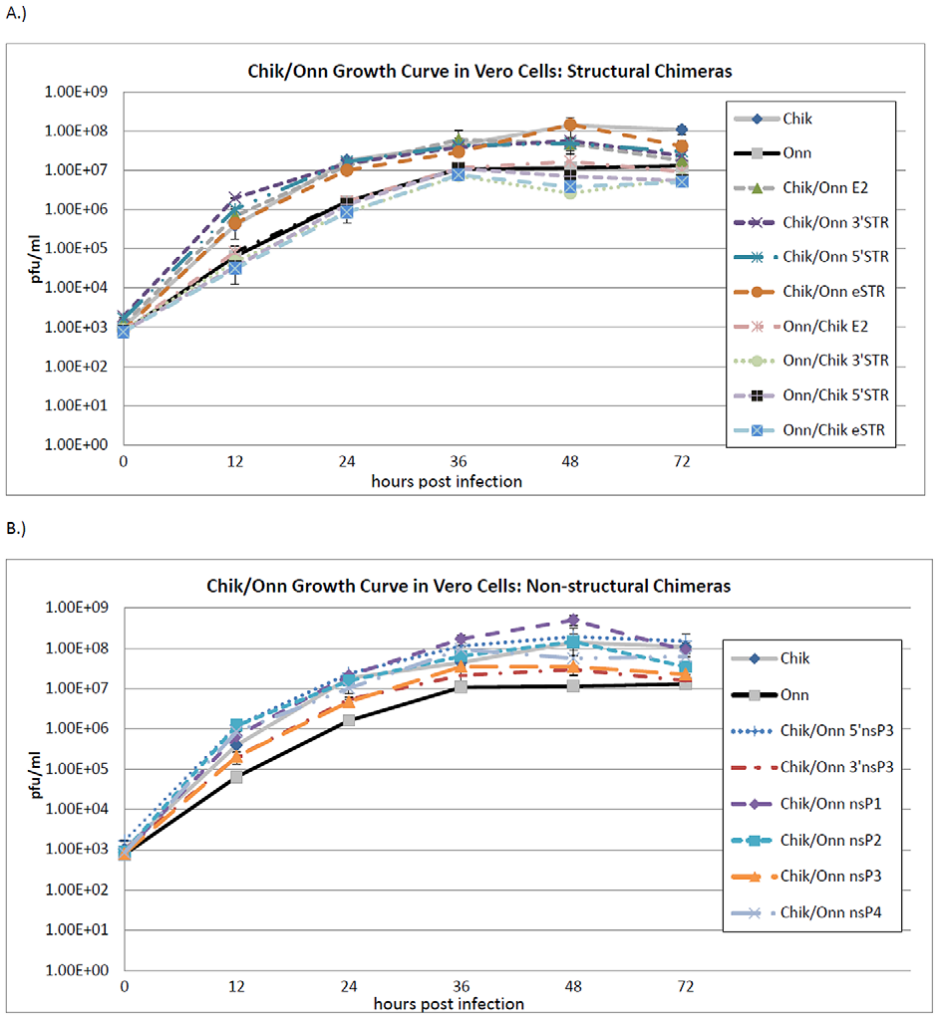
Growth curves for CHIKV and ONNV chimeras in Vero cells after infection at 0.1 MOI. (A) Structural region chimeric viruses, (B) Non-structural region chimeric viruses.

Understanding the involvement of viral elements in vector specificity is critical for eventual control of vector-borne viruses. This study built upon previously established disparate infectivity patterns for two closely related alphaviruses, CHIKV and ONNV, in *An. gambiae* [5], [6] and infection rates with the parental viruses generated from our full-length infectious clones were concordant with those previously reported. By day 12, up to 91% of *An. gambiae* mosquitoes were infected with ONNV, whereas a maximum of only 6% were infected with CHIKV. These values are similar to previously published work [6]. With this highly significant difference (p<0.0001) between the two viruses, characterization of individual viral gene substitutions was likely to reveal which elements were involved in mosquito infection. Prior to initiating these experiments with chimeric viruses in *An. gambiae*, viral replication (and not just persistence of input virus) within both cell culture and in the mosquito was confirmed. Cell culture growth curves of all of the chimeras were performed in Vero cells to confirm that all viruses were indeed replication competent and replicated in a manner similar to their parental viruses (Figure 3). The structural change viruses all replicated efficiently and replication was virtually identical among all chimeras sharing non-structural genes. In general, those viruses with the ONNV non-structural genes grew to peak titers of 10^6.5^–10^7^ pfu/mL while those with CHIKV non-structural genes had peak titers of 10^7.5^–10^8^ pfu/mL. All non-structural chimeric viruses grew similarly well, rapidly increasing in titer from 1000 pfu/mL to 10^7^–10^8.5^ pfu/mL. No consistent statistical differences were observed among the non-structural substitution viruses.

**Figure 4:**
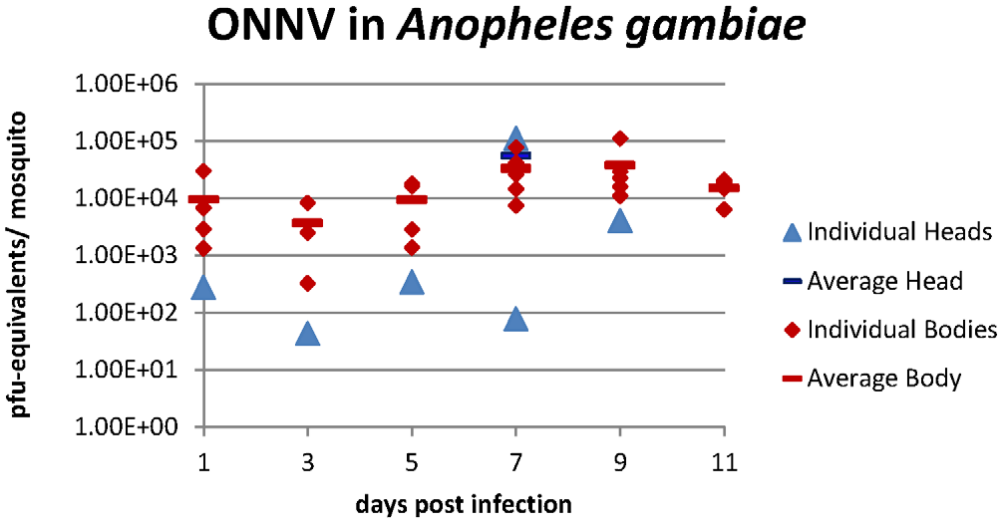
ONNV RNA quantification in *An. gambiae* by reverse-transcriptase real-time PCR. Five mosquitoes were analyzed for each timepoint and samples with positive values were plotted. Average values (represented by horizontal bars) were only calculated when more than one positive sample was present for that time point.

The quantity of ONNV RNA present in individual mosquito bodies and heads through 11 days post-infectious feed adhered to the expected pattern of decrease during the extrinsic incubation period followed by a rise in virus replication at later time points as determined by qRT-PCR. Moreover, after 5 days post infection, the five mosquito bodies tested at each of the subsequent time points had more RNA copies than could have been initially imbibed in the blood meal indicating replication of the virus was indeed occurring (Figure 4).

Nine unique chimeric hybrids of CHIKV and ONNV were constructed using convenient restriction enzyme sites to produce substitutions in the structural region of the viral genome and to examine the contribution of each of these specific regions to virus-vector specificity (Figure 1). Six additional non-structural chimeric viruses were also constructed using a novel type II restriction enzyme cloning strategy to examine the broader genome with respect to ONNV's unique vector specificity for *An. gambiae* mosquitoes (Figure 2).

Having confirmed viral replication and infections rates of parental ONNV in *An. gambiae*, infection and dissemination rates with each of the 15 chimeric viruses were determined. Each time a virus was generated through *in vitro* transcription for this study, it was sequenced completely prior to use in an infectious feed. Mosquitoes containing replicating virus in the body, as shown by CPE analysis, were defined as being positive for viral infection. Mosquito heads were analyzed separately from bodies to determine dissemination rates.

Each chimeric virus constructed from the parental CHIKV genome maintained a CHIKV-like infection profile (<10% infection rate), with one exception. When allowed to feed on a blood meal containing approximately 5.5 log_10_ PFU/ml of CHIK/ONN nsP3 virus, 63.5% (n = 85) of mosquitoes had replicating virus when harvested on day 8 post infection (Figure 2). None of the ONNV substitutions made to the structural regions of the CHIKV parental genome produced infection results deviating from those seen with the complete CHIKV parental genome (Figure 1). Three of the 5 chimeric viruses constructed from the parental ONNV genome retained ONNV-like infection rates at day 8 in *An. gambiae*, while the remaining two viruses showed significantly lower infection rates. Only 11.1% (n = 135) of mosquitoes feeding on ONN/CHIK 3′STR and 53.2% (n = 77) of ONN/CHIK 5′STR were shown to be infected at day 8. Infection rates for mosquitoes sacrificed at days 4, 11, or 12 corroborate day 8 results (data not shown).

Dissemination rates for each of the viruses in *An. gambiae* was very low and all were comparable for both day 8 and day 11 samples. Only 5 viruses showed any dissemination (figures 1 and 2): parental ONN.AP3,Onn/Chik E2, Onn/Chik eSTR, Onn/Chik 3′nsP4-5′C, Chik/Onn 3′STR. The rest of the viruses showed no dissemination.

## Discussion

A panel of 15 chimeric viruses were developed here to study specific elements of the ONNV genome and to determine which of these regions are necessary for ONNV to infect *An. gambiae* mosquitoes. As CHIKV virus primarily infects *Aedes* species and ONNV primarily infects *Anopheles* species, these two closely related viruses provide an ideal opportunity to study these viral genetic determinants of infection. This study is the first to look at the importance of ONNV non-structural proteins in *An. gambiae* infection. Of the ten CHIKV-backbone chimeras constructed and tested, only the one containing ONNV nsP3 produced infection rates closer to parental ONNV than to the parental CHIKV. The ability of ONNV nsP3 to up-regulate infection rates so substantially shows that ONNV nsP3 is the main determinant of ONNV vector specificity for *An. gambiae*. Interestingly, the reciprocal chimeric virus (full length-ONNV with the CHIKV nsP3) was not able to be rescued from cDNA in either mammalian or insect cells. This would further suggest that nsP3 plays a critical role in viral replication that is distinct in these two closely related viruses that exhibit 81% and 72% amino acid and nucleotide identity respectively in nsP3. That nsP3 should be found to be essential to infection is especially interesting given the fact that the precise functions of this protein are not fully defined. It is required for the correct formation and localization of replication complexes and does provide essential functions in both minus strand and subgenomic RNA synthesis, but specific mechanisms are not yet resolved [21]–[23].

To further add to the intrigue of this protein, it has been shown that some members of the alphavirus family actually contain inserts of foreign genetic material within nsP3. An eight amino acid sequence from the carboxyl-terminus of CHIKV nsP3 maps to a putative zinc finger protein in *Ae. aegypti*, the main vertebrate vector for that virus [26]. In Semliki Forest virus, a 7 amino acid sequence corresponds to elements found in a wide-range of cellular proteins [26]. Numerous other examples of what may be inserts of foreign genetic material been shown by sequencing nsP3 from the following alphaviruses: CHIKV, eastern equine encephalitis virus, Semliki Forest virus, and Venezuelan equine encephalitis virus [26].

Alphavirus nsP3 can be clearly divided into two distinct domains. The macro domain, or amino-terminal region, is highly conserved, not just among alphaviruses but also among coronaviruses, hepatitis E virus, rubella virus and even cellular proteins [27], [28]. The carboxyl-terminus domain of the alphavirus nsP3 is highly variable in size and sequence and is devoid of any predicted secondary structure [29], [30]. Chimeric viruses were constructed using the natural division between the conserved and non-conserved regions of nsP3 to engineer two additional chimeric viruses to determine if the region conferring specificity to *An. gambiae* could be attributed solely to either of the distinct domains within nsP3. Interestingly, the addition of just the carboxyl-terminus of ONNV nsP3 did produce a small, although not a statistically significant, increase in infection rates as compared with parental CHIKV in *An. gambiae*.

The carboxyl-terminus of nsP3, which has been subject to rapid alteration during alphavirus evolution, may also be involved in the optimization of replication in diverse host cell types [29]. Studies with Sindbis showed that deletions in the carboxyl-terminus rendered mutants defective at initiating a productive infection, generating plaques in mosquito cells at only 1–2% the efficiency of the parental virus [31]. A recent study noted a carboxyl-terminus, proline-rich sequence motif, the PIPPPR motif, shared by many alphavirus nsP3 proteins and demonstrated that even a single mutation in this region of Semliki Forest virus or Sindbis virus greatly impaired RNA synthesis by disrupting binding with host cell amphiphysins [32]. It is possible that this motif also modulates ONNV vector specificity since ONNV and CHIKV do differ from one another by one amino acid in this PIPPPR region. Attenuated virulence and reduced rates of RNA synthesis and virus replication were also seen in vertebrate cells with Semliki Forest virus mutants lacking some portion of the carboxyl-terminus of nsP3 [33]. Yet, studies in mammalian cell lines showed that a 34 amino acid deletion in this region of nsP3 in Venezuelan equine encephalitis had no detectable effect on replication [34]. Collectively, these studies support the current finding that nsP3 can be vital for productive infection, but in a manner that is host and virus specific.

Another interesting characteristic of the carboxyl-terminus of nsP3 is that it is phosphorylated at multiple serine and threonine residues [35], [36]. The role of this phosphorylation is not exactly clear, except that it does seem to modulate the efficiency of minus-strand RNA synthesis [37], [38]. Determination of the exact mechanisms of this modulation and the mechanisms for the host-specific effects seen with nsP3 mutants in this and other studies would be extremely valuable information and allow for design of further studies. Furthermore, our studies show that an intact ONNV nsP3 is required for ONNV-like infection rates, and that dividing the region either disrupts a vital interaction between the two or removes an element necessary for *An. gambiae* infection. The former seems more probable since substituting CHIKV for either half of ONNV nsP3 results in infection rates not significantly different from rates with parental CHIKV.

While molecular determinants residing in nsP3 did turn out to be the most dramatic finding of our study, we did also examine the structural regions of the genome. Previously published studies by another group had suggested that all of the viral structural proteins are necessary for ONNV to infect *An. gambiae* mosquitoes [21]. Our study was able to provide critical fine tuning to this conclusion. In our experiments with CHIKV-backbone chimeras containing various regions of the ONNV structural proteins, each maintained parental CHIKV-like infection profiles despite containing portions of the ONNV genome. In fact, even an intact ONNV structural region was not sufficient for infection of *An. gambiae*, as shown with the chimera CHIK/ONN eSTR (Figure 1). The reciprocal chimeras, substituting sections of CHIKV structural regions for the like section of ONNV structural genes, in most cases, did not greatly reduce mosquito infection rates. The notable exceptions were in the chimeras that divided the ONNV structural region in half. Both ONN/CHIK 5′STR and ONN/CHIK 3′STR were significantly less infectious to *An. gambiae* than was parental ONNV. However, since the reciprocal chimeras, CHIK/ONN 5′STR and CHIK/ONN 3′STR, did not show up-regulated infection rates, the drop in infection with the chimeric viruses is likely due to disruption of one or more virus-virus or virus-host interactions.

In alphaviruses, the extreme 3′ terminus of the genome, just preceding the poly(A) tail, has a sequence which is highly conserved among all alphaviruses and which is absolutely required for efficient virus replication [39], [40]. This 19-nucleotide sequence is identical in CHIKV and ONNV so this could not have played a role in the decreased infection rates seen with ONN/CHIK 3′STR. However, studies using Sindbis mutants with large deletions in the 3′non-translated region (NTR) have shown that the rest of the 3′ NTR is also important for virus replication in a host-specific manner [40]. ONNV is 156 additional nucleotides shorter in the 3′NTR when compared to CHIKV; this size difference alone could result in conformational changes resulting in the inability of the virus to interact with itself or with host proteins. Of note is the design of our eSTR and 3′STR structural clones, which contain the indicated structural region as well as the 3′ NTR from the non-parental virus (Figure 1); this design is different from those previously described [21] and may suggest the possibility of multiple interactions within the proteins or gene sequences of the virus itself that may have a minor role in the overall ability of a chimeric virus to replicate within the mosquito. It is further possible that the differences in CHIKV and ONNV conserved sequence elements [41] are sufficient to undercut RNA stability, resulting in greatly reduced mosquito infection patterns.

Studies with chimeric viruses must be viewed in the overall context of the virus' life cycle. When substitutions are made to construct chimeric viruses, numerous aspects of the virus-host interactions and virus-virus regulatory functions can be disrupted resulting in reduced infection rates. Reduced infection rates may be a direct consequence of missing the essential genomic region or may be an indirect result of disrupting an essential regulatory interaction. Conversely, when the addition of a specific region increases mosquito infection rates, we must conclude the region itself to be essential for infection. Interestingly, because there was such a low dissemination rate of all viruses within this study, elements involved in dissemination throughout the mosquito may be distinct from those important in initial infection. However, this study has shown that ONNV nsP3 is directly responsible for ONNV infection of *An. gambiae*. There are also numerous interactions within nsP3 itself, within the two halves of the structural region, and possibly the 3′ NTR which, when disrupted, can eliminate mosquito infection.

## Supporting Information

Figure S1 **Illustration of exact nsP3 substitution made to create CHIK/ONN nsP3.**
(TIFF)

Figure S2 **Construction of CHIKV nsP3 receiving plasmid.** PCR primers were designed to generate two amplicons flanking the DNA insertion sites and extend outward to include unique restriction enzyme sites and inward to include a unique type II restriction site. Amplification with these primers, subsequent digestion with PciI/SacI or EcoRI/SacI, followed by a 3-part ligation produce a pUC-based vector containing CHIKV sequence flanking the site where ONNV nsP3 will later be inserted.
(TIFF)

Figure S3 **Amplifying ONNV nsP3.** PCR primers were designed to amplify the desired DNA insert, with the addition of type II restriction enzyme sites to the termini. Type II sites were oriented such that they will be removed upon later digestion.
(TIFF)

Figure S4 **Expanded sequence of assembled CHIKV nsP3 receiving plasmid (top). Termini of ONNV nsP3 amplicon (bottom).** The lines indicate the cut sites for the type II restriction enzymes.
(TIFF)

Figure S5 **Products produced after digestion with appropriate type II restriction enzymes.** These products were ligated to build the CHIKV/ONNV nsP3 cassette plasmid.
(TIFF)

Figure S6 **Construction of final clone, Chik/Onn nsP3.** CHIKV/ONNV nsP3 cassette plasmid and pCHIK.b were digested with SpeI and AvrII. The resulting products were ligated to generate the final clone with the complete ONNV nsP3 gene replacing the like gene in CHIKV.
(TIFF)

Protocol S1 **Methods for construction of gene specific clones.**
(DOCX)
