# Supplementary figures and images for: O'nyong nyong Virus Molecular Determinants of Unique Vector Specificity Reside in Non-Structural Protein 3
Source: PLoS Negl Trop Dis. 2013 Jan 24;7(1):e1931. doi: 10.1371/journal.pntd.0001931 (PMC3554527; doi:10.1371/journal.pntd.0001931)

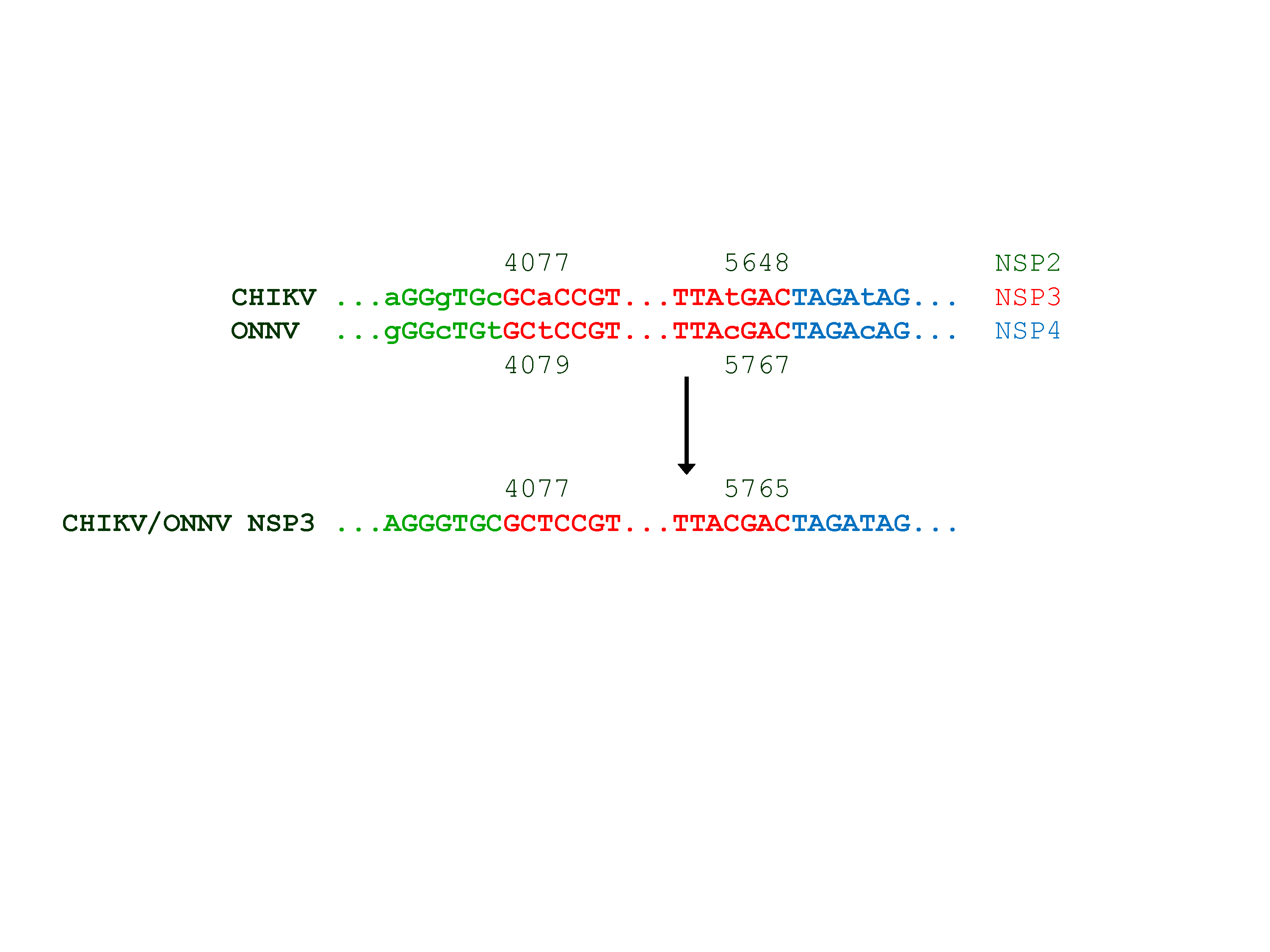

Supplement: Figure S1 — Illustration of exact nsP3 substitution made to create CHIK/ONN nsP3. (TIFF) [file pntd.0001931.s001.tiff]

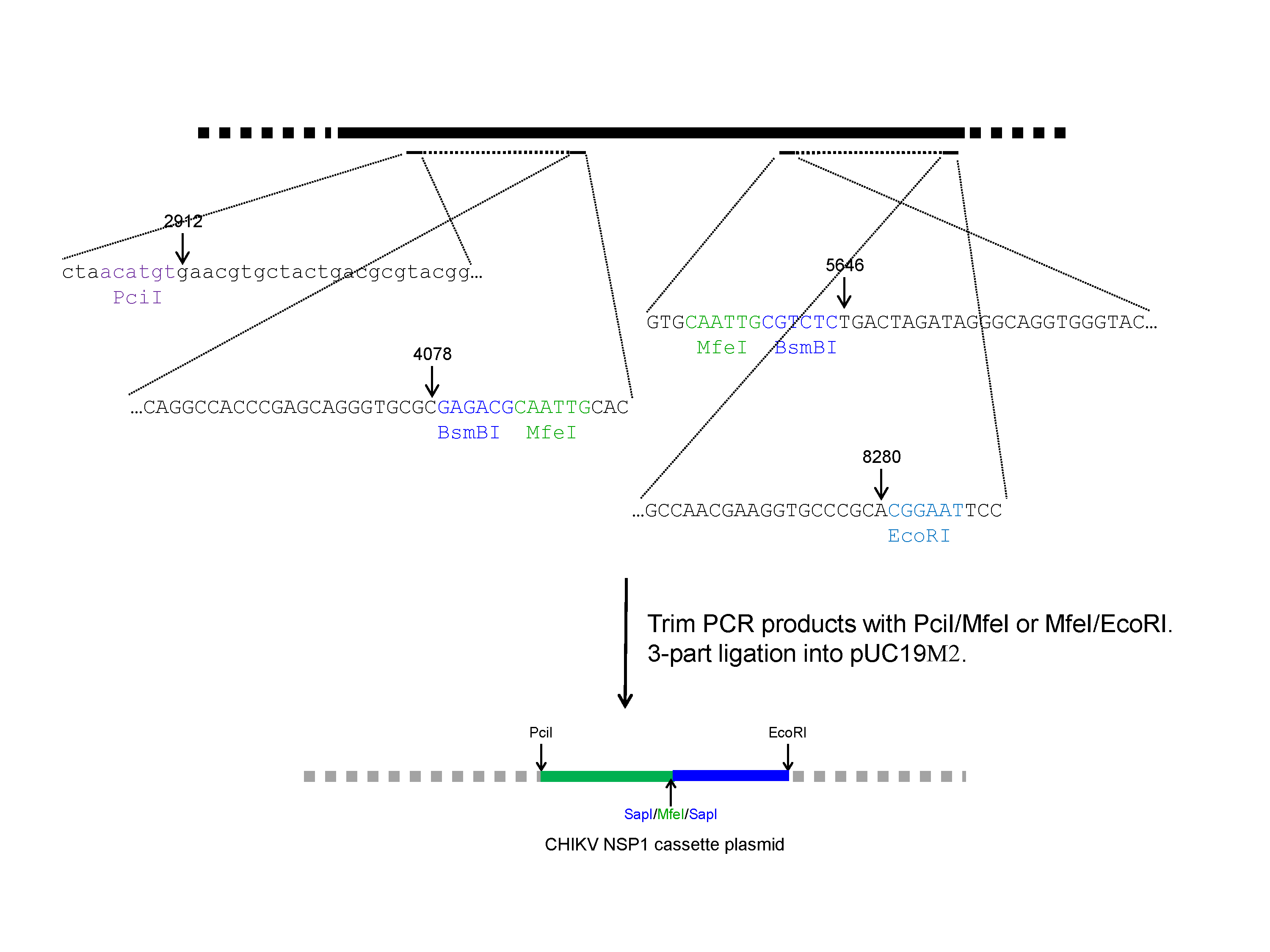

Supplement: Figure S2 — Construction of CHIKV nsP3 receiving plasmid. PCR primers were designed to generate two amplicons flanking the DNA insertion sites and extend outward to include unique restriction enzyme sites and inward to include a unique type II restriction site. Amplification with these primers, subsequent digestion with PciI/SacI or EcoRI/SacI, followed by a 3-part ligation produce a pUC-based vector containing CHIKV sequence flanking the site where ONNV nsP3 will later be inserted. (TIFF) [file pntd.0001931.s002.tiff]

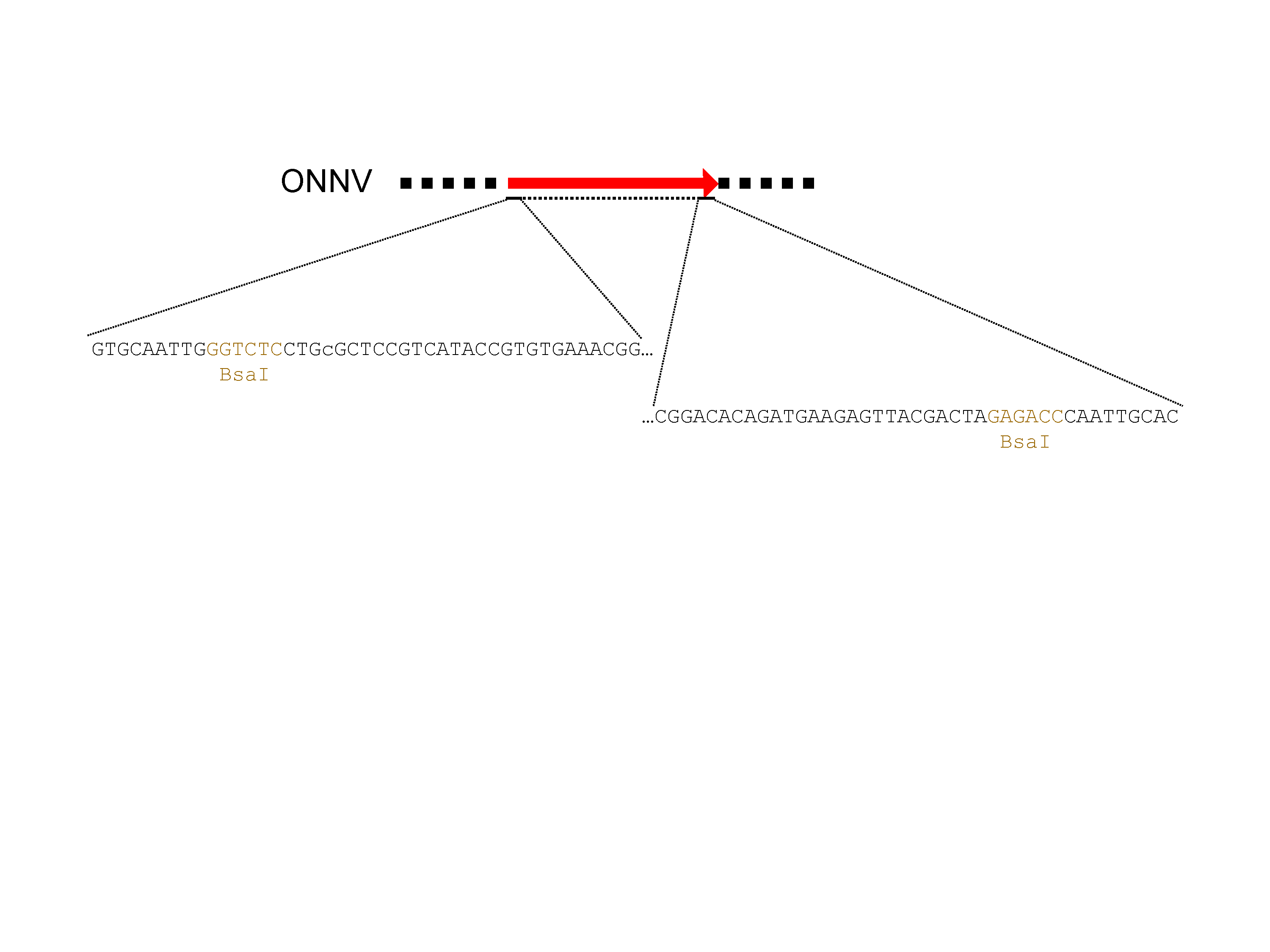

Supplement: Figure S3 — Amplifying ONNV nsP3. PCR primers were designed to amplify the desired DNA insert, with the addition of type II restriction enzyme sites to the termini. Type II sites were oriented such that they will be removed upon later digestion. (TIFF) [file pntd.0001931.s003.tiff]

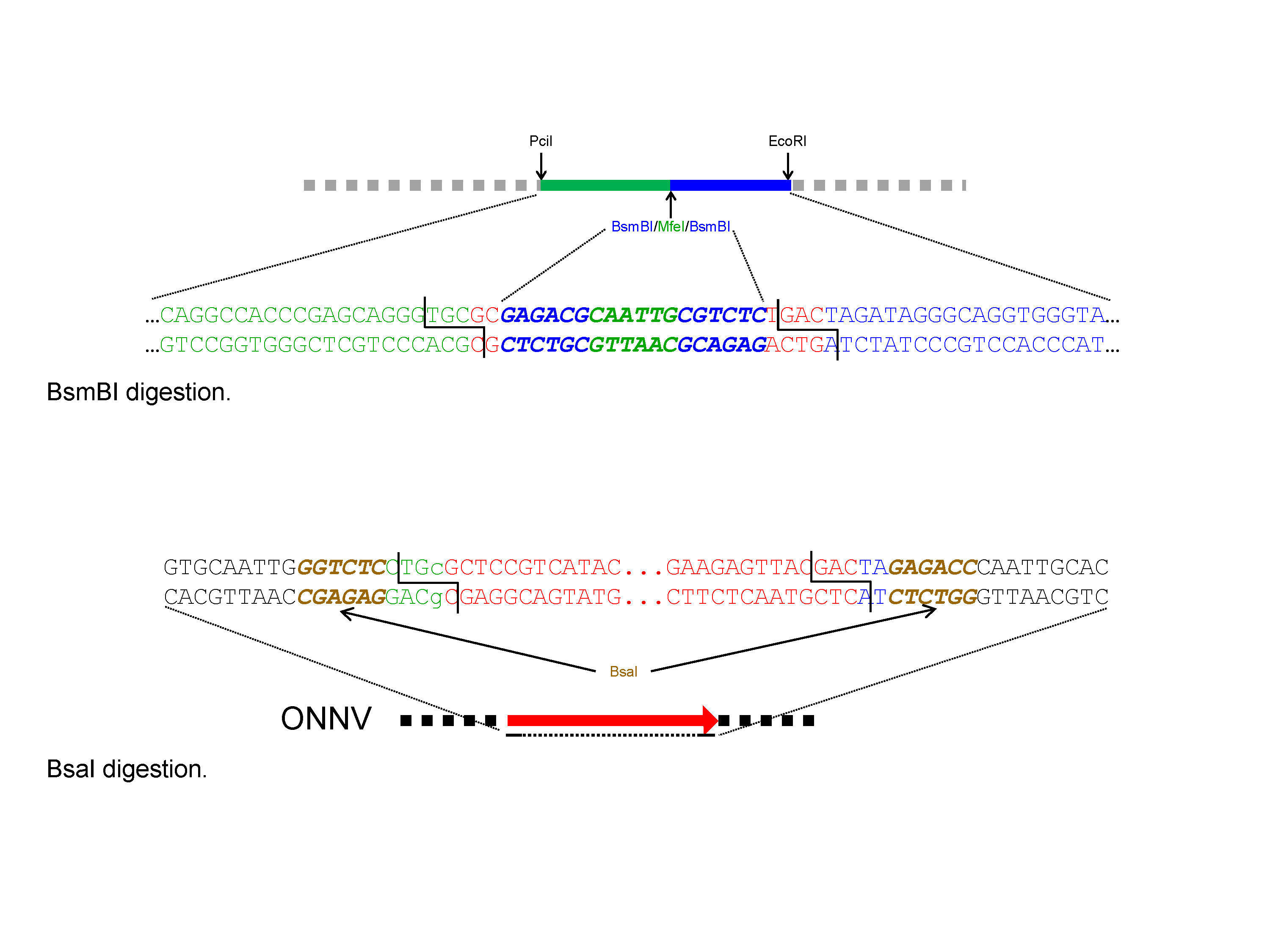

Supplement: Figure S4 — Expanded sequence of assembled CHIKV nsP3 receiving plasmid (top). Termini of ONNV nsP3 amplicon (bottom). The lines indicate the cut sites for the type II restriction enzymes. (TIFF) [file pntd.0001931.s004.tiff]

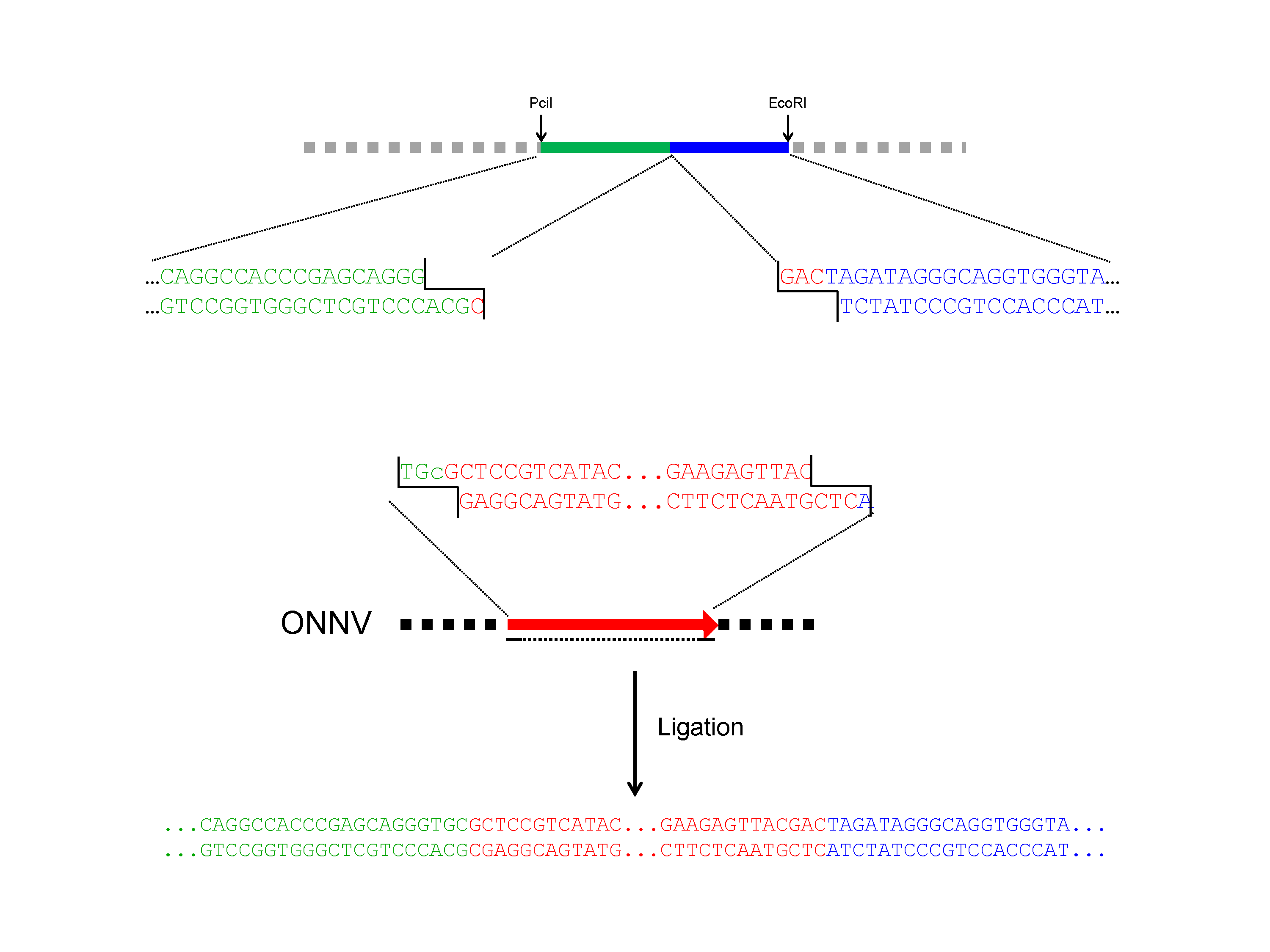

Supplement: Figure S5 — Products produced after digestion with appropriate type II restriction enzymes. These products were ligated to build the CHIKV/ONNV nsP3 cassette plasmid. (TIFF) [file pntd.0001931.s005.tiff]

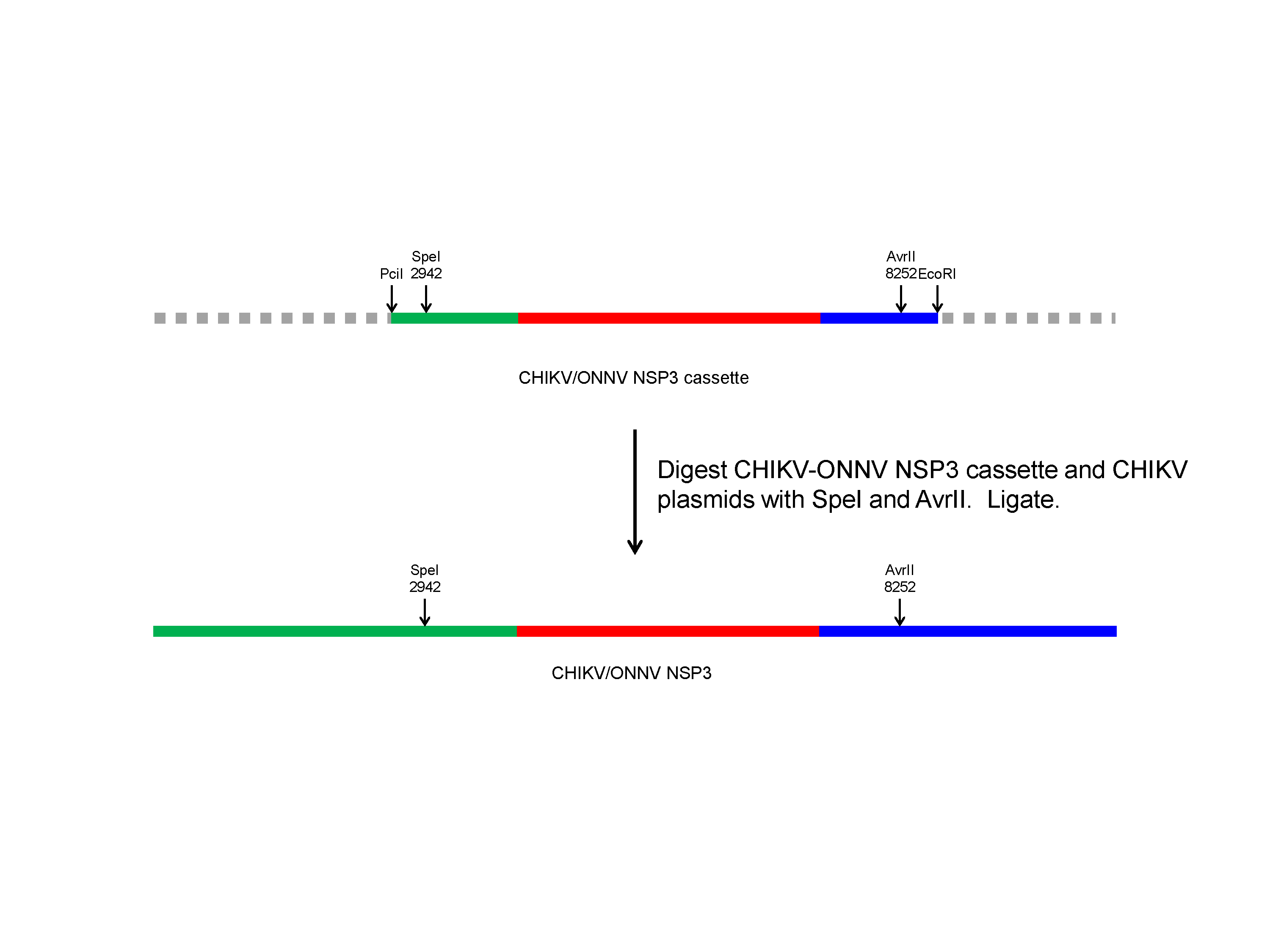

Supplement: Figure S6 — Construction of final clone, Chik/Onn nsP3. CHIKV/ONNV nsP3 cassette plasmid and pCHIK.b were digested with SpeI and AvrII. The resulting products were ligated to generate the final clone with the complete ONNV nsP3 gene replacing the like gene in CHIKV. (TIFF) [file pntd.0001931.s006.tiff]
