# Supplementary material for: O'nyong nyong Virus Molecular Determinants of Unique Vector Specificity Reside in Non-Structural Protein 3
Source: PLoS Negl Trop Dis. 2013 Jan 24;7(1):e1931. doi: 10.1371/journal.pntd.0001931 (PMC3554527; doi:10.1371/journal.pntd.0001931)
Supplement: Protocol S1 — Methods for construction of gene specific clones. (DOCX) [file pntd.0001931.s007.docx]

**Supplemental Protocol**

This cloning process was adapted from Blakqori and Weber (J Virol 79: 10420-10428) to allow the construction of CHIKV/ONNV chimeras that exactly matched the respective portions of both of the parental genomes that were under consideration. The following example describes our technique for inserting ONNV nsP3 in the place of CHIKV nsP3 (CHIK/ONN nsP3; Figure S1). Throughout these figures, nsP2, nsP3, and nsP4 sequences are represented in green, red, and blue, respectively.

*Construction of CHIKV nsP3 receiving plasmid.*

Unique restriction sites were identified in the parental plasmid (i.e. pCHIK.b) that flank the region being substituted. pCHIK.b has a unique SpeI site at nucleotide 2942 and a unique AvrII site at nucleotide 8252. The unique XbaI site at nucleotide 7440 is dam methylated and so was not chosen for use. Importantly, these sites also do not occur in the foreign DNA that will be substituted for the like parental region.

PCR primers were designed to generate two amplicons flanking the DNA insertion sites that extend outward to the unique sites described above (Figure S2). The inward facing primers each add a convenient restriction site, in this case PciI and EcoRI for cloning into a pUC-based vector. It is important to the design that neither of these restriction sites occur elsewhere in the amplicon to which it is attached. The outward facing primers contain a linker restriction site not present in either amplicon (SacI) and type II restriction enzyme recognition sites oriented such that when the amplicon was cut by the enzyme, the recognition site was removed (BsmBI). The type II recognition site should not be present elsewhere in the amplicons, the vector into which the amplicons will be cloned, or in the foreign DNA (in this case ONNV nsP3) to be later inserted. If this occurs, as it does in this example, alternate type II restriction enzymes leaving similar overhangs can be employed.

DNA was amplified and digested with PciI and SacI or EcoRI and SacI, as appropriate. Digested amplicons were cloned into a previously mutated (to remove the BsmBI sites) pUC19 vector, pUC19M2, using a 3-part ligation. Successful ligation generated the CHIKV nsP3 receiving plasmid.

*Construction of ONNV nsP3 DNA to be inserted into cassette.*

Primers were designed to amplify the DNA to be inserted, adding type II restriction enzyme recognition sites to the termini (Figure S3). These sites were again oriented inward such that digestion with the enzyme resulted in the removal of the recognition site. The sites were placed so that the resulting overhang was complementary to the overhang left by the type II enzymes described in Figure S2. Note that this required the inclusion of a mismatch in the nsP2 terminal nucleotide (lowercase “c”), so that the final clone would maintain fidelity to the parental sequence. As ONNV nsP3 contains a BsmBI recognition site, BsaI was selected for this amplicon. Both BsmBI and BsaI leave a 4-base 5’ overhang.

*Construction of the CHIK/ONN nsP3 cassette plasmid.*

The expanded sequence of the assembled CHIKV nsP3 receiving plasmid insertion site and the termini of the ONNV nsP3 amplicon are shown in Figure S4. The lines indicate the cut sites for the type II restriction enzymes. Digestion with the appropriate type II restriction enzymes generated the products shown in Figure S5. These products were ligated to build the CHIKV/ONNV nsP3 cassette plasmid.

*Construction of CHIK/ONN nsP3.*

CHIK/ONN nsP3 cassette plasmid and pCHIK.b were digested with SpeI and AvrII. The resulting products were ligated to generate CHIK/ONN nsP3 (Figure S6).
